# Incidence and Severity of Lymphoedema following Limb Salvage of Extremity Soft Tissue Sarcoma

**DOI:** 10.1155/2011/289673

**Published:** 2011-11-20

**Authors:** Daniel Friedmann, Jay S. Wunder, Peter Ferguson, Brian O'Sullivan, David Roberge, Charles Catton, Carolyn Freeman, Neil Saran, Robert E. Turcotte

**Affiliations:** ^1^Division of Orthopedic Surgery, McGill University Health Centre, Montreal, QC, Canada H3G 1A4; ^2^Musculoskeletal Oncology Unit Division of Orthopedic Surgery, Mount Sinai Hospital, Toronto, ON, Canada M5G 1X5; ^3^Department of Surgical Oncology, Princess Margaret Hospital, Toronto, ON, Canada M5G 2C4; ^4^Department of Radiation Oncology, Princess Margaret Hospital, Toronto, ON, Canada M5S 3S2; ^5^Department of Radiation Oncology, McGill University Health Centre, Montreal, QC, Canada H3G 1A4

## Abstract

*Background and Purpose*. Lymphoedema is a serious complication following limb salvage for extremity soft tissue sarcomas (STSs) for which little is known. We aimed to evaluate its incidence, its, severity and its associated risk factors. *Material and Method*. Patient and tumor characteristics, treatment modalities and complications and functional outcomes (MSTS 1987, TESS), and lymphoedema severity (Stern) were all collected from prospective databases. Charts were retrospectively abstracted for BMI and comorbidities. *Results*. There were 289 patients (158 males). Mean age was 53 (16–88). Followup ranged between 12 and 60 months with an average of 35 and a median of 36 months. Mean BMI was 27.4 (15.8–52.1). 72% had lower extremity tumors and 38% upper extremity. Mean tumor size was 8.1 cm (1.0–35.6 cm). 27% had no adjuvant radiation, 62% had 50 Gy, and 11% received 66 Gy. The incidence of lymphoedema was 28.8% (206 none, 58 mild, 22 moderate, 3 severe, and 0 very severe). Mean MSTS score was 32 (11–35) and TESS was 89.4 (32.4–100). Radiation dose was significantly correlated with tumor size > 5 cm (*P* = 0.0001) and TESS score (*P* = 0.001), but not MSTS score (*P* = 0.090). Only tumor size > 5 cm and depth were found to be independent predictors of significant lymphoedema. *Conclusion*. Nine percent of STS patients in our cohort developed significant (grade ≥ 2) lymphoedema. Tumor size > 5 cm and deep tumors were associated with an increased occurrence of lymphoedema but not radiation dosage.

## 1. Introduction

Soft tissue sarcomas (STSs) comprise a group of rare malignant tumors occurring most commonly in the extremities [1, 2]. Preferred treatment for patients with STS is limb preservation surgery usually in combination with adjuvant preoperative or postoperative radiation therapy [1, 3–7]. This treatment carries a significant risk of functional disability and reduced quality of life [6, 8], and up to 50% of patients live with significant long-term disability [9].

Studies that have compared preoperative to postoperative external-beam radiotherapy (EBRT) as part of limb sparing surgery for patients with extremity STS have shown that, while preoperative EBRT allows the use of lower doses and smaller treatment fields, such an approach is associated with increased risk of acute wound healing complications [3, 6, 10]. Postoperative EBRT typically requires the use of higher radiation doses and larger target volumes and is associated with increased late radiation-related morbidity [3, 11]. One important complication which has been given very limited attention thus far is secondary lymphoedema. 

Lymphoedema is swelling that generally occurs in the limbs, or less commonly in visceral and axial structures, due to an accumulation of protein-rich lymph fluid in the interstitial tissues [12, 13]. In patients with STS, lymphatic injury may result from surgical disruption of lymph nodes, lymphatic or major blood vessels. Alternatively, the lymphatic system may be damaged by radiotherapy leading to fibrosis and compromised lymph transport [12]. Patients presenting with lymphoedema secondary to treatment for STS are prone to developing recurrent infections and skin changes such as hyperkeratosis and papillomatosis [14]. Beyond the physical symptoms and signs, lymphoedema may also be associated with significant psychological and functional morbidities such as poor body image leading to anxiety and depression [15]. 

Lymphoedema in the upper extremities has been studied extensively in the breast cancer population. The frequency of occurrence is extremely variable ranging from 3% to 83%, although generally accepted to be approximately 30% [16–19]. Several predisposing factors have been identified, most importantly axillary surgery (lymph node dissection) and axillary radiotherapy [15, 17, 19, 20]. Other factors which may also influence the risk of lymphoedema in the breast cancer population include the stage at diagnosis, systemic therapies (chemotherapy or hormonal therapy), age, body mass index, hypertension, history of infection, and pretreatment education regarding lymphoedema and preventive self-care activities [2, 15, 17, 21, 22].

Few studies have addressed the question of lymphoedema in patients with STS, which occurs most commonly in the lower extremities [2, 4, 8]. Previous soft tissue sarcoma series reported an incidence of lymphoedema of 30% while others recorded significant lymphoedema (grade ≥2) in 19% [4, 8, 18, 23]. High biologically effective dose (BED), radiation field length >35 cm and lower extremity location were identified as positive risk factors for the development of chronic oedema. 

The objective of this study was to identify the incidence and the severity of lymphoedema, and to evaluate the potential risk factors in patients with extremity soft tissue sarcoma who have undergone limb preservation surgery with or without adjuvant external beam radiotherapy.

## 2. Methods

Our prospective tumor database served to identify patients who had undergone surgical management of extremity STS at the Montreal General Hospital, Montreal, Quebec, and the Mount Sinai Hospital, Toronto, Ontario, between 2000 and 2007. All patients selected for inclusion in this study had prospective collection of lymphoedema severity rating at an interval of at least 1 year following treatment. Lymphoedema severity was evaluated using Stern's Rating Scale for Edema. Stern's scale is a subjective, physician-rated measure with scores ranging from 0 to 4 (Table 1) [25]. Functional outcomes were assessed using the Musculoskeletal Tumor Society Rating scale (MSTS) and the Toronto Extremity Salvage Score (TESS). The MSTS is a clinician-rated scale which evaluates pain, joint range of motion, strength, joint stability, joint deformity, overall function, and general acceptance of the treatment; the score ranges from 0 to 35 [24]. The TESS is a patient-rated measure developed specifically for patients undergoing limb salvage surgery for bone and soft tissue sarcomas which evaluates difficulty performing daily activities [26]. All three outcomes were collected simultaneously at a mean interval of 35 months from treatment (range 12–60). Patients' demographics, tumor characteristics, and treatment-related variables were prospectively collected. Charts were also retrospectively abstracted for body mass index (BMI) and to identify medical comorbidities including the occurrence of thrombophlebitis.

The association between size of the tumor and the likelihood of having significant lymphoedema (grade 2 or 3) was modeled using logistic regression. Tumor size was categorized as small or large with large representing tumors 5 cm or greater, and the small category was used as the reference for the analysis. The following variables were included as independent variables to obtain adjusted effects: age was a continuous variable, and sex, BMI, upper versus lower extremity, whether or not a lymph node dissection was performed, radiation, and smoking were categorical variables with male sex, BMI <30 kg/m^2^, upper extremity, no node dissection, no radiation, and nonsmoker coded to serve as reference categories. Initially, depth of the tumor was also included as a variable; however, seeing as there were no cases of lymphoedema in the superficial group, this had to be removed from the analysis. Regression coefficients were exponentiated to determine the odds ratio (OR) of significant lymphoedema compared to nonsignificant lymphoedema for a large tumor as compared to a small tumor. The log-likelihood ratio test was used to assess the significance of the association fitted by the model, and individual regression estimates are tested by Wald statistics for significance by assessing the null hypothesis that the regression estimates are equal to zero. A *P*-value of 0.05 was used as the cut-off for significance. All statistical analyses were performed using SPSS 19 (IBM, Armonk, NY).

## 3. Results

 288 patients met the inclusion criteria of whom 55% were male. The mean age was 53 years (range 16–88). Mean body mass index (BMI) was 27.43 (range 15.8–52.1). 73% of patients presented with lower extremity tumors. Specific tumor locations, in order of frequency, were as follows: quadriceps (*N* = 95), adductor (*N* = 43), shoulder (*N* = 38), elbow/forearm (*N* = 36), hamstring (*N* = 26), knee (*N* = 17), ankle/foot (*N* = 15), buttock (*N* = 12), and hand (*N* = 6). Average tumor size was 7.4 cm (range 1.0–35.6). Tumor histology, in order of frequency, was as follows: MFH (*N* = 74), liposarcoma (*N* = 64), leiomyosarcoma (*N* = 30), synovial (*N* = 28), fibrosarcoma (*N* = 25), DFSP (*N* = 12), MPNST (*N* = 10), osteosarcoma (*N* = 10), rhabdomyosarcoma (*N* = 2), and other (*N* = 23). Patient demographics and tumor and clinical characteristics are given in Table 2. Radiation therapy was administered preoperatively (50 Gy) for 184 patients, postoperatively (66 Gy) for 21 patients, and both pre- and postoperatively (50 + 16 Gy) for 7 patients. 76 patients received no radiation therapy. Table 3 shows treatment modalities in patients with none or minimal lymphoedema and in those with more severe limb swelling.

Posttreatment lymphoedema was identified in 29% (*N* = 83) of patients. Mild lymphoedema (grade 1) was observed in 58 patients, moderate (grade 2) in 22, and severe (grade 3) in 3. Patients with moderate and severe lymphoedema were grouped together and compared with a second group consisting of patients with either mild or no lymphoedema. The incidence of significant posttreatment lymphoedema (i.e., grade ≥2) was 9%. Seven patients developed post-operative thrombophlebitis, and all of them had subsequent lymphoedema rated as none (6) or mild (1). We found 16 patients who underwent lymph node dissection (5.5%). Among these 2 received 66 Gy, 10 received 50 Gy, and 4 received no radiation. Out of those 16 patients, 3 patients had significant lymphoedema, 6 showed mild lymphoedema, and 7 had none; thus 13 of the 16 did not demonstrate significant lymphoedema. The radiation dose administered was significantly correlated with tumor size >5 cm (*P* = 0.0001) and tumor depth (*P* < 0.001), but not tumor location (i.e., upper versus lower extremity, *P* = 0.334). Higher radiation dosage was also significantly correlated with lower TESS score (*P* = 0.001), but not MSTS score (*P* = 0.090). 

Univariate analysis identified significant correlations between the severity of lymphoedema and radiation dose (50 versus 66 Gy; *P* = 0.010), tumor size (>5 cm; *P* = 0.011), and deep location (*P* = 0.001). We found no significant correlation between the severity of lymphoedema and tumor location (upper versus lower extremity) or lymph node dissection. We also found no significant association between the incidence of lymphoedema and the body mass index (BMI), hypertension, or smoking.

In the group of patients with deep tumors, the incidence of significant lymphoedema was 12%. There were no cases of lymphoedema in the superficial tumor group (*χ* ^2^ = 10.168, *P* = 0.001) irrespective of the amount of radiation received. The unadjusted OR of significant lymphoedema (grade 2 or 3) in large (5 cm or greater) as compared to small (less than 5 cm) tumors was 19.7 (*P* = 0.004; 95% CI: 2.6 to 148.8) and the adjusted OR was 12.4 (*P* = 0.02; 95% CI: 1.5 to 100.9) (Table 4). The computed log-likelihood ratio test was 98.99 with 8 degrees of freedom and *P* = 0.006 suggesting that at least one of the regression coefficients is different from zero. These results show that lymphoedema was strongly associated with the size of the tumor.

## 4. Discussion

There have been very few reports addressing the incidence and severity of lymphoedema following modern management of soft tissue sarcoma of the extremities. Reasons for the rare occurrence of sarcoma, the need for large number of patients, and significant disparities among tumor, location, and management in contrast to breast or gynaecologic cancers. This series consists of a large number of patients treated in a multidisciplinary setting with prospective data collection including lymphoedema severity assessment and function outcomes. We recorded an overall incidence of 29% of lymphoedema in sarcoma of the extremities which is identical to a previous report [8].

We elected to use Stern's Rating Scale of lymphoedema severity as it is relatively simple and has been used as part of our combined prospective data collection for years. However, even in experienced hands, this classification remains subjective and somewhat imprecise and no intra- and interobserver reliability tests have been performed to demonstrate its validity. Additionally, it has been suggested that qualitative measurements of lymphoedema may minimize its true incidence [27]. Despite the need for quantitative tools to assess lymphoedema severity [28], recent proposals addressing this topic have only led to minor variations and Stern's Rating Scale is very similar to those endorsed by the National Cancer Institute (NCI) and the Radiation Therapy Oncology Group (RTOG) [12, 29].

There are important limitations in the current study. The number of patients enrolled in this study remains relatively small to provide for sufficient power in subset analyses. All patients treated for extremity soft tissue sarcoma were not systematically included. Lymphoedema grading was mandatory for inclusion in the study, and an unknown number of cases may have been excluded if recording was omitted. Patients who died or were lost to followup within the first year following treatment were also excluded. Risk factors such as smoking habits, body mass index, and medical comorbidities were also not prospectively and systematically collected for all patients leading to softer conclusions about their role in the occurrence of lymphoedema. Timing for the recording of lymphoedema may also be important as it may appear or get worse over many years [27, 28]. With median and average follow-up periods of 3 years from the index procedure it was likely that we captured most of the severe cases. We could not identify whether there were patients who underwent lymphoedema treatments prior to or at the time of recording lymphoedema severity. Although thrombophlebitis, an important cause of chronic extremity swelling, was not systematically recorded, we did not identify any significant swelling in the 7 patients who suffered a recognized thrombotic event. Moreover our study was likely underpowered to detect the expected increased incidence of chronic swelling in lower limb tumor or following lymph nodes dissection. Perhaps the most important limitation to the conclusions of this work was the discrepancy and the relative small number of patients including those that received 66 Gy of radiotherapy demonstrating a clear bias toward neoadjuvant radiotherapy. The timing of radiation therapy in the treatment of soft tissue sarcoma remains controversial. In a prospective randomized study, it was shown that neoadjuvant radiotherapy led to a postsurgical wound complication rate of 34% in the preoperative group compared to 17% when radiation was administered post-operatively [18]. Functional status, as per the Toronto Extremity Salvage Score, was found to be identical in both groups but, at a minimum 2-year followup, patients treated with higher dose postoperative radiotherapy tended to have significant fibrosis, joint stiffness, and edema, all of which correlated with worse functional outcomes [4]. Similar findings were previously reported [8]. Although univariate analysis identified a significant relationship between the occurrence of significant lymphoedema and a total dose of 66 Gy, radiotherapy was not found to be associated with lymphoedema using multivariate analysis. Despite this, one must be careful in concluding no association as the number of patients in this study are limited for such a conclusion. The possible benefit of preoperative radiation in minimizing some of the late treatment effects such as lymphoedema remains to be demonstrated. These potential benefits need to be balanced against the risk of acute wound healing complications on an individual basis within the multidisciplinary treatment setting. The impact of wound complication of the incidence of lymphoedema also remains unknown. We did not record and compare the radiotherapy target volume. However, in our centers with well-established multidisciplinary teams, it is logical to expect that the radiotherapy target volume would correlate with tumor volume. Thus, as for large tumors, a larger volume of tissue irradiated would likely lead to increased incidence of late treatment-related morbidity, including lymphoedema. addition, intensity modulated radiotherapy (IMRT) has become routine in recent years because of improved dosimetry, specifically improved sparing of bone and joints as well as of skin and other noninvolved tissues from high dose exposure. All of these factors may be expected to lead to reduced late treatment-related morbidity in future patients [30, 31]. The limited number of patients and heterogeneity of radiation modalities meant that we could not perform useful comparisons.

The value of a postoperative boost of radiotherapy following standard 50 Gy preoperative radiotherapy has been put into question recently and may be unnecessary to prevent local recurrence [32]. Others have suggested that lower than traditional postoperative radiotherapy doses reduce the occurrence of chronic sequelae without compromising local control [33]. Although a lower dose of radiotherapy may be safe we cannot recommend it for the prevention of lymphoedema. 

It remains unclear how function and quality of life relate to the incidence and severity of lymphoedema. In the current study, TESS scores were significantly lower in patients who received a higher dose of radiation therapy (*P* = 0.0001), but MSTS scores did not correlate with radiation dosage. Functional results from previous Canadian randomized clinical trial found that both TESS and MSTS scores correlated with skin fibrosis, joint stiffness, and lymphoedema, and these were dependant on the radiotherapy regimens [4]. Although lymphoedema most likely impacts physical function, our findings do not suggest that worse functional outcome is mainly the result of lymphoedema. 

The effect of treatment on chronic lymphoedema also remains unclear. Some have reported an overall improvement in health-related quality of life (HRQOL) using the Nottingham Health Profile Part-1 (NHP-1) as the main outcome measure [34]. The treatment resulted in significant changes in the physical domains (e.g., mobility) but no significant change was noted in the emotional or psychological domains. 

Lymphedema can be a troublesome and important complication of limb salvage treatment for STS. The overall incidence of lymphedema in our study was 29% with significant (grade 2 or more) lymphedema occurring in 9% of patients. Risk factors for lymphedema included depth of tumor and tumors >5 cm in size. Interestingly, radiotherapy was not found to be significantly associated with lymphedema. It has been reported that cancer patients are not always informed about lymphoedema symptoms or management and that the uses of prevention strategies could be improved [17]. The occurrence of lymphoedema might be minimized through increased awareness, education, and therapy. Prospective trials are needed to determine the potential effect of pretreatment education and prophylactic interventions on the incidence of lymphoedema in the STS population.

## Figures and Tables

**Table 1:** Stern's Rating Scale for Edema [24].

Score	Rating
0	None
1	Mild (but definite swelling)
2	Moderate
3	Severe (considerable swelling)
4	Very severe (skin shiny and tight ± skin cracking)

**Table 2:** Patient demographics and tumor and clinical characteristics in relation to lymphoedema severity.

Variable (*N*)	Lymphoedema <2	Lymphoedema ≥2
Age at surgery (288)		
Mean	52	58
Range	16–86	19–88
Gender (288)		
Male	144	14
Female	119	11
Presenting status (288)		
Primary	249	24
Local recurrence	12	1
Unplanned	2	0
Extremity (288)		
Upper	59	22
Lower	204	3
Tumor size (288) [cm]		
Mean	7.1	11.4
Range	1.0–35.6	4.5–20.5
Tumor depth (288)		
Superficial	78	0
Deep	185	25
AJCC Stage (272)		
IA	42	0
IB	18	0
IIA	63	8
IIB	52	2
IIC	12	1
III	62	12

**Table 3:** Lymphoedema severity versus wound closure, tissue resected, and comorbidities.

Variable (*N*)	Lymphoedema <2	Lymphoedema ≥2
Surgical closure (288)		
Primary	194	20
Free flap + STSG	14	3
STSG only	46	0
Other	9	2
Tissues resected		
Skin/subcutaneous	176	21
Muscle	122	21
Bone	20	2
Nerve	27	3
Vessels	18	2
Comorbidities		
Myocardial infarction	37	2
Coronary artery disease	22	4
Hypertension	86	7
Deep vein thrombosis	7	0
Renal failure	6	0
Diabetes type II	17	3
Ulcer	4	2

**Table 4:** Logistic regression coefficients with Corresponding *P* values, odds ratios, and 95% confidence intervals of the odds ratios.

Variable	Regression coefficient	Std. error	Wald (*df *= 0)	*P*	Odds ratio	95% Confidence Interval	
						for odds ratio	
						Lower bound	Upper bound
Constant	−7.06	1.77	15.964	0.000	0.001		
Age (years)	.021	0.018	1.460	0.227	1.022	0.987	1.058
Sex (male)	−0.330	0.550	0.360	0.548	0.719	0.244	2.113
Extremity (Lower)	1.463	0.870	2.829	0.093	4.320	0.785	23.765
BMI (≥30)	−.671	0.738	0.826	0.363	0.511	0.120	2.173
Tumor size (large)	2.515	1.071	5.517	0.019	12.372	1.516	100.932
Lymph node Dissection	1.705	1.017	2.811	0.094	5.504	0.750	40.407
Radiation	0.219	0.712	0.095	0.758	1.245	0.309	5.019
Smoking	0.505	0.556	0.826	0.364	1.657	0.558	4.923

BMI: body mass index. Age was a continuous variable. Sex, extremity, BMI, tumor size, lymph node dissection, radiation, and smoking are categorical values with reference categories as follows: male sex, upper extremity, BMI <30 kg/m^2^, tumor size <5 cm, no node dissection, no radiotherapy, and nonsmoker.
